# Supplementary material for: Preparation, Identification and Application of β-Lactoglobulin Hydrolysates with Oral Immune Tolerance
Source: Foods. 2023 Jan 9;12(2):307. doi: 10.3390/foods12020307 (PMC9857568; doi:10.3390/foods12020307)
Supplement: Supplementary file 1 [file foods-12-00307-s001.zip › foods-1978127-supplementary.pdf]

Preparation, Identification and Application of β-Lactoglobulin Hydrolysates with Oral Immune Tolerance

Linghan Tian [1], Qianqian Zhang [1], Yanjun Cong [1,*] and Wenjie Yan [2]

[1] Beijing Higher Institution Engineering Research Center of Food Additives and Ingredients, College of Food and Health, Beijing Technology and Business University, Beijing 100048, China
[2] College of Biochemical Engineering, Beijing Union University, Beijing 100023, China
* Correspondence: congyj@th.btbu.edu.cn

Supplementary Information

Table of Contents

Table S1 Prediction of T cell epitope regions of β-LG by IEDB---------------------2

Table S2 Prediction of T cell epitope regions of β-LG by NetMHC(II)/Pan------3

Figure S1 A schematic diagram of pre-oral exposure using β-LG hydrolysate and intragastric sensitization--4

Figure S2 DH of β-LG hydrolysates---5

Table S1. Prediction of T cell epitope regions of β-LG by IEDB.

Allele	Starting amino acid	Predictive T epitope	Adjusted-rank
HLA-DRB1*08:01	110	VLDTDYKKYLLFCMENSAE	4.52
HLA-DRB1*08:02	26	LDIQKVAGTWYSLAMAASDIS	11.84
HLA-DRB1*08:09	154	KALKALPMH	1.62

Table S2. Prediction of T cell epitope regions of β-LG by NetMHC(II)/Pan.

Allele	Starting amino acid	Predictive T epitope	Core	Score	Bind level
HLA-DRB1*08:01	32	AGTWYSLAMAASDIS	YSLAMAASD	0.30	WB
	83	AQKKIIAEKTKIPAVFKIDALNENKV	IAEKTKIPA	0.48	WB
HLA-DRB1*08:01	13	GAQALIVTQTMKGLDIQ	LIVTQTMKG	0.43	WB
	55	LRVYVEELKPTPEGD	YVEELKPTP	0.33	WB

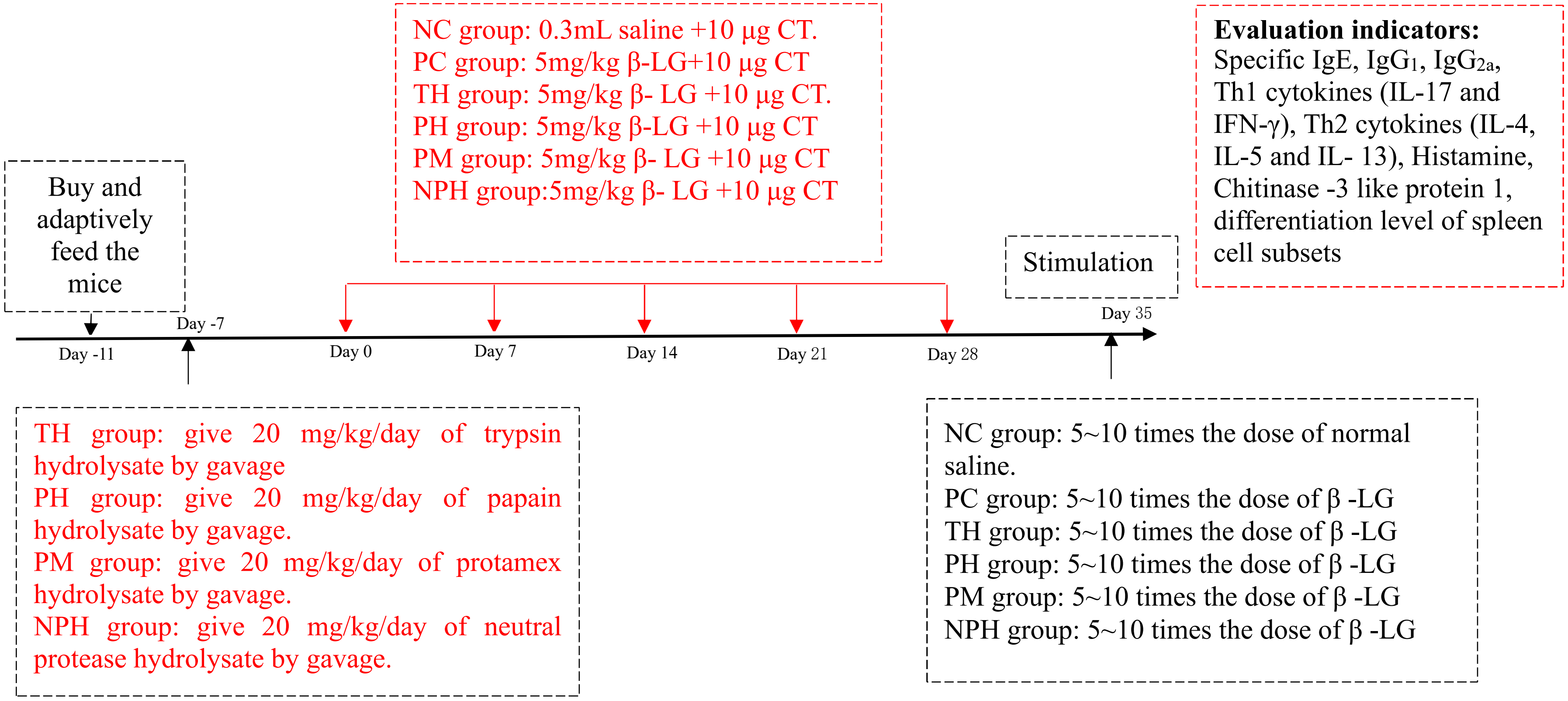

Figure S1. A schematic diagram of pre-oral exposure using β-LG hydrolysate and intragastric sensitization.

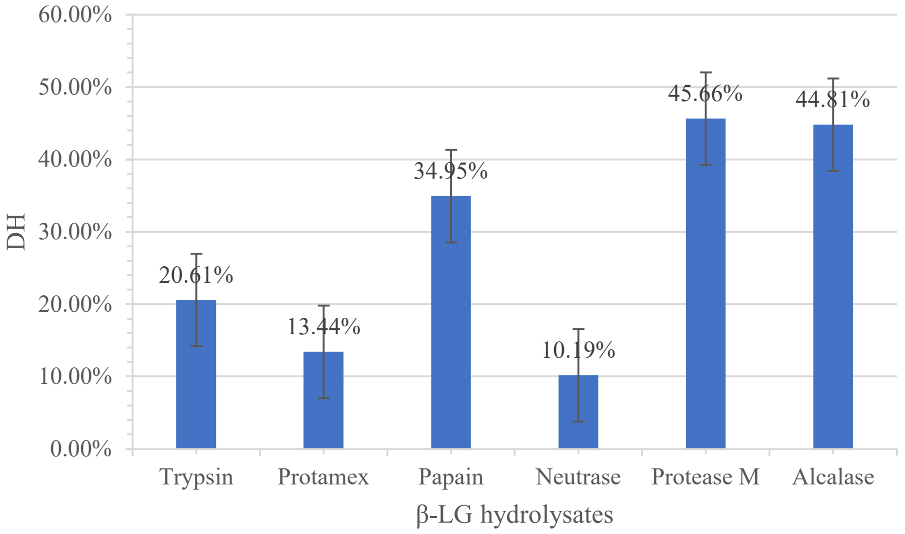

Figure 2. DH of β-LG hydrolysates.
